# Timed Up-and-Go Dual-Task Testing in the Assessment of Cognitive Function: A Mixed Methods Observational Study for Development of the UDDGait Protocol

**DOI:** 10.3390/ijerph17051715

**Published:** 2020-03-05

**Authors:** Ylva Cedervall, Anna M. Stenberg, Hanna B. Åhman, Vilmantas Giedraitis, Fredrik Tinmark, Lars Berglund, Kjartan Halvorsen, Martin Ingelsson, Erik Rosendahl, Anna Cristina Åberg

**Affiliations:** 1 Department of Public Health and Caring Sciences, Geriatrics, Uppsala University, SE-75185 Uppsala, Sweden; 2 Department of Geriatrics and Rehabilitation Medicine, Falu Hospital,. SE-70182 Falun, Sweden; 3 The Swedish School of Sports and Health Sciences, SE-11433 Stockholm, Sweden; 4 Department of Anatomy, Physiology and Biochemistry, Swedish University of Agricultural Science, SE-75007 Uppsala, Sweden; 5 Systems and Control, Department of Information Technology, Uppsala University, SE-75105 Uppsala, Sweden; 6 Department of Mechatronics, Campus Estado de Mexico, Tecnologico de Monterrey, Monterrey 64849, NL, Mexiko; 7 Department of Community Medicine and Rehabilitation, Physiotherapy, Umeå University, SE-90187 Umeå, Sweden; 8 School of Education, Health and Social Studies, Dalarna University, SE-79188 Falun, Sweden

**Keywords:** screening, memory assessment, feasibility, study protocol, dementia diagnosis, cognitive impair

## Abstract

New methods to screen for and identify early-stage dementia disorders are highly sought after. The purpose of this pilot study is to develop a study protocol for a dual-task test aimed at aiding the early detection of dementia disorders. We used the Timed Up-and-Go (TUG) test, which is a mobility task involving starting in a sitting position, standing up, walking three meters to cross a line on the floor, turning around, walking back and sitting down again. We combined TUG with the verbal task of naming different animals. Pilot study participants were 43 individuals with and without established dementia diagnoses who attended a clinic for memory assessment. Video-recorded test performances were systematically analysed. Deviant test performances concerning the interplay between test administration and participants’ responses to the assessment instructions were revealed and led to refinements being made to the final study protocol. Exploration of the dual-task test outcome measures in a sub-sample of 22 persons, ten with and twelve without dementia, indicated that step-length and number of named animals after the turning point of the dual-task test might constitute appropriate measures for examining this kind of sample. We concluded that the refined study protocol is feasible for testing individuals undergoing initial memory assessments and healthy controls. Follow-up studies with larger samples are being carried out and will bring new knowledge to this area of research. It may also provide an opportunity for further studies exploring possibilities for broad clinical implementation.

## 1. Introduction

Dementia disorder is a complex syndrome and chronic illness, characterised by a global and irreversible cognitive decline that is severe enough to undermine the performance of activities of daily living (ADL). The causes are physical changes in the brain, most commonly due to Alzheimer’s disease (AD) [1]. Dementia impairs multiple higher functions, including memory, orientation, comprehension, learning, calculation, judgment, and verbal functioning. Other, less severe cognitive impairment diagnoses such as mild cognitive impairment (MCI) and subjective cognitive impairment (SCI) may represent transitional stages between normal ageing and dementia. The diagnosis of MCI is defined as a cognitive impairment that is greater than what is to be expected in relation to the person’s age and education but does not affect ADL [2], whereas SCI has been regarded as a possible forerunner of MCI [3]. Moreover, a pre-dementia “motor cognitive risk syndrome” characterised by slow gait and cognitive complaints has been introduced, and the need for accurate concepts for the identification of modifiable risk factors among people with this syndrome has been stressed [4,5].

It has been estimated that approximately 75% of people with dementia disorders in the world have not yet been diagnosed, and the majority of people with dementia disorders are diagnosed in the later stages of the illness [6]. This implies a significant treatment gap. Screening for the early identification of cognitive impairment and dementia disorders is, therefore, crucial for the implementation of measures that will ameliorate symptoms and increase the quality of life for afflicted patients and their relatives [6,7]. Currently, there is no simple way to diagnose dementia early on. The Mini Mental State Examination (MMSE) [8] is the best-established screening test for cognitive function and is widely used in clinical settings and research. A recent study has shown that a three-step screening program, with MMSE as the starting point, could enhance the diagnosis of dementia disorders and, also, increase healthcare utilization related to diagnosed dementia disorders among community-dwelling people aged 60 years and above [9]. However, the MMSE does not provide measurements of cognitive capabilities that are completely reliable. The floor and ceiling effects for different groups, as well as the lack of evaluation of executive functions, have been noted as weaknesses [10]. In a Cochrane review, it was concluded that MMSE is not suitable as a stand-alone single-administration test classification for the identification of individuals with MCI that could progress to a dementia disorder [11]. As such, MMSE cannot be used alone as a diagnostic instrument and is unsuited for early diagnosis. On the other hand, gait disturbances have been associated with cognitive impairment in an increasing number of studies [12,13,14]. It has been concluded that there is no causal link between cognitive and motor dysfunction, but findings reflect the fact that the control of such functions stems from overlapping neuronal networks in the central nervous system [13]. Studies that have demonstrated a connection between the control of gait and cognitive functions have supported the proposal to incorporate gait testing into memory assessment, since gait tests may have the potential to support early identification of cognitive impairment [15].

Studies have also shown that reduced cognition is associated with poorer performance of the Timed Up-and-Go test (TUG) [16,17]. TUG is a well-established, uncomplicated, and quickly administrated test of one movement sequence: starting from a sitting position in an armchair, standing up and walking three meters, turning around, walking back to the chair, and sitting down again [18]. The original outcome is the time it takes to complete the test. TUG is well tested for the assessment of gait and mobility and has shown good reliability for people with AD [19]. It has been indicated that both reduced gait speed and TUG performance could predict future cognitive decline [16].

Individuals with dementia disorders also demonstrate marked changes in their gait when completing tasks that require dual-task attention: for example, gait combined with a simultaneous verbal task, compared with cognitively healthy individuals from the same age group [20,21,22]. Therefore, gait tests combined with a simultaneous verbal task have been suggested to have the potential of serving as a support in the early identification of cognitive impairment and dementia disorders [13,23]. The conclusions drawn are that adequate gait testing may be helpful for the detection of early stages of cognitive impairment, as well as an increased risk of falling associated with cognitive decline. Research is therefore increasingly directed towards the new paradigm in which mobility and cognition are viewed as interrelated and that there is a need for an integrated assessment approach that includes both these domains [24]. Such an approach can be achieved through dual-task testing, which has been shown to be more sensitive to cognitive decline than single-task gait performance measures [25,26]. Gait parameters derived from dual-task tests have additionally been suggested as markers for detecting dementia [20,27,28]. One suggested explanation for these results is that dual-task testing can serve as a brain stress test with the potential of revealing degrees of cognitive impairment at a subclinical stage [29]. The advantages of such tests are that they can be simply conducted in a clinical setting, the performance/results of the test are barely affected by education level, and the gait test is ecologically valid since it relates to ADL [5,13]. Thus, TUG testing with dual-task performance could be regarded as a potentially valuable clinical tool for screening and enabling treatment planning for patients with cognitive impairment [30].

Previous TUG dual-task research, regarding differentiating patients with dementia disorders, cognitive impairment diagnoses, and healthy controls, has focused on time scores [31,32]. The TUG as a component in dual-task testing has also been used to investigate gait parameters, where stride frequency appeared to correlate with the level of cognitive function [33]. Further research, however, is required to explore results from TUG dual-task performance among people with different levels of cognitive functioning as well as its capacity to predict future cognitive decline and future dementia diagnosis.

### Research Objectives 

With the purpose of providing a screening test that can aid the early detection of dementia disorders, we have developed a TUG dual-task test (TUGdt) procedure. This test involves TUG being performed simultaneously with the verbal task of naming different animals, which we have previously used in a study with individuals with mild AD [32]. For this new purpose, we are planning to evaluate the prognostic power of the TUGdt test’s outcomes through an initiated large-scale longitudinal cohort trial named the Uppsala Dalarna Dementia and Gait study (UDDGait).

In an attempt to respond to the currently highlighted need for more publications in this field and the sharing of feasibility work concerning non-randomised studies [34], we are hereby reporting on the processes of the pilot study for feasibility evaluation, preparatory work and the development of the UDDGait protocol.

The overall aim of this pilot study is to develop and refine a feasible UDDGait protocol. Specific objectives are to:

1. Identify adaptations to improve the content and structure of the study protocol in cooperative consensus processes with expert clinicians and researchers in the area of memory assessment.
2. Identify possibilities for refinement of the assessment procedures based on investigations of uncertainties concerning the interplay between TUGdt administration and participants’ responses to the assessment instructions by the use of video-recorded test performances.
3. Explore potentially useful TUGdt outcome measures based on TUGdt assessments of a small sample of individuals attending a specialist clinic for memory assessment.

## 2. Material and Methods

### 2.1. Study Design

A mixed methods approach was adopted in this observational pilot study. The current article follows the main features of Consolidated Standards of Reporting Trials (CONSORT) extensions to pilot trials (https://www.equator-network.org/reporting-guidelines/consort-2010-statement-extension-to-randomised-pilot-and-feasibility-trials/), as suggested for pilot and feasibility studies in preparation for non-randomised cohort studies [34].

### 2.2. Ethical Approval and Patients’ Consent to Participate

An effort was put on adapting all information to the participants to make it clear, understandable and objective, and to provide a good base for informed consent. If the participant wished, we also informed their relatives. The agreement for participation includes a possibility to choose whether or not retouched (to preserve personal integrity) video-recordings of the data collection are allowed to be shown in relation to scientific presentations. This was not a requirement for participation. Only video-recordings from individuals who have provided consent for us to use them are shown as part of the results presentation. The Regional Ethical Review Board in Uppsala has approved the UDDGait, including the current pilot study, Dnr. 2014/068.

### 2.3. Pilot Setting and Participating Patients

For the pilot study, participants were recruited among patients who were scheduled for a memory assessment visit or a re-visit at the specialist clinic of Uppsala University Hospital, Uppsala, Sweden, during a period of four weeks. The exclusion criteria were inability to walk three meters back and forth or to rise up from a sitting position, indoor use of a walker, need of an interpreter to follow instructions given in Swedish, current or recent (within the last two weeks) hospitalization, and disapproval from either a treating physician or a close relative.

**Figure 1:**
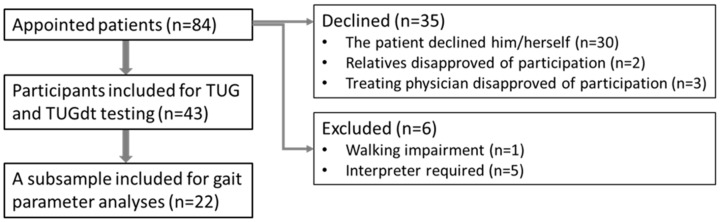
Flow chart for the inclusion in the pilot study.

During the study period, 84 memory assessment appointments were completed. Thirty of these patients declined study participation. The most common reasons for declining were “want to focus on my medical appointment”, “cannot manage it”, and “do not want to”. Another five patients declined due to disapproval from their physician or a relative. These disapprovals were motivated by the risk of study participation disturbing the medical appointment. Six patients were excluded in accordance with the exclusion criteria: need of an interpreter (n = 5), and walking impairment (n = 1) (Figure 1).

The remaining 43 patients were included. Their median age was 74 years (range: 58–87 years), 26 were men, and 25 had an established dementia disorder diagnosis (DD) while 18 had no established dementia diagnosis (NoDD). Among the latter, nine had MCI, and nine needed further assessment before a diagnosis could be established (Table 1). We used convenience sampling, based on non-deviant TUGdt performance according to the qualitative video analyses (see below) for a sub-sample of participants for the exploration of TUGdt outcome step length (SL) extracted from the video data (Table 1).

### 2.4. Pilot Test Procedures, Data Collection and Analyses

Collection of data for our three specific pilot objectives: (i) identification of adaptations to improve the study protocol, (ii) identification of possibilities for refinement of the assessment procedures, and (iii) exploration of potentially useful TUGdt outcome measures, were carried out in somewhat overlapping processes, described below.

Two of the authors (Y.C. and A.C.Å.) are both researchers and physiotherapists (Pt) specialised in geriatrics, with experience from clinical work and research on dual-task testing in memory assessment [21,32]. They created the basis for the pilot study protocol. Refinement of this protocol involved the assessor (AS), who is a Pt and MSc-student with experience in geriatric care. She carried out all the data collection in terms of clinical tests. A checklist to standardise the TUGdt procedures was developed and used and supervised training sessions with expert feedback (by the responsible Pts) were accordingly carried out.

Face-to-face consensus meetings were held with a team of clinicians and researchers (including a neuropsychologist, nurses, occupational therapists, social workers and specialist physicians) working at the study site. The goals were to spread information about the study, facilitate cooperation for the data collection, and receive input regarding methodology. For evaluation from a patient’s point of view, one older person without cognitive impairments was recruited at a nearby out-patient ward, tested, and interviewed about the structure, test order, instructions, time, and any difficulties experienced during the testing. This latter process resulted in only minor refinements being made.

Collection and processing of patients’ data were blinded in relation to diagnoses. Demographic data was collected through oral reports from the participants and, if he/she so wished, also from their relatives. All testing followed the same procedure and was carried out according to standardised written protocols in the memory clinic setting, as mentioned above. Participants’ heights (with shoes on) were measured and established clinical tests of cognition and motor functions were performed in accordance with standard protocols and were mainly used for descriptive purposes, i.e., hand grip strength of the dominant hand using a dynamometer (Patterson Medical, Sutton-in-Ashfield, UK), mobility by the General Motor Function Assessment Scale [35,36], cognitive functions using the Mini Mental State Examination (MMSE) [8], and the Clock Test [37]. The diagnoses were collected from the medical records in connection to the data entry.

#### 2.5.1. Timed Up-and-Go and Timed Up-and-Go dual-task tests

A standardised set up was used for the TUG and TUGdt tests, including video documentation. The TUGdt consisted of TUG combined with the verbal attention-demanding task of naming different animals, which challenges semantic memory and executive function [38,39]. The task of naming animals is based on a well-established verbal fluency test [40], which is commonly used for assessing semantic memory and has been used as a component in various dual-task tests [24,41].

The starting position of the participant was sitting in an armchair placed three meters in front of a marked line on the floor. They were allowed to use their hands to help them stand up. Instructions were given verbally and testing was initiated by a trial TUG test alongside the assessor before the actual testing was started. Then the TUG test was performed followed by the TUGdt test. Instructions for both tests were, as stipulated on the checklist: Walk at a self-selected, comfortable speed, pass the marked line, turn around and walk back to the chair, sit down again. The TUGdt test had the additional instruction: Name different animals while walking, if you cannot think of any animals, continue walking and finish the mobility task. In other words, participants were asked to prioritize walking over the verbal task during the TUGdt test.

The TUG and TUGdt tests were timed by the assessor with a stopwatch to the nearest 0.01 s. from the participant rising up (his/her back leaving the chair’s backrest) to sitting down (his/her posterior touching the chair’s seat). To capture the TUG and TUGdt test outcomes concerning the mobility and verbal performances as well as SL, these tests were recorded using two cameras (Sony NEX-5T, Sony Corporation, Tokyo, Japan). One was placed two metres in front of the line where the subjects turned, and the other was four metres to the side of the line.

#### 2.5.2. Analyses

Systematic qualitative analyses of all the TUGdt video recordings (n = 43) were carried out by Y.C. and any uncertainties were checked and discussed with A.C.Å. This resulted in a systematic presentation of identified deviant mobility and verbal performances related to the TUG test’s sub-sequences and in general: (i) Initiation including rising up from the chair, (ii) Walking 3 m to the line, (iii) Line crossing and turning, (iv) Walking back 3 m to the chair, (v) Sitting down on the chair (Table 2A,B). Viewing and listening to the video recordings allowed the total number of animals recited to be counted. This also enabled repeated viewings and hence validation of this data collection. Unusual/indistinct words could be investigated to determine if they denoted an animal or not. Since the qualitative analysis of the videos indicated that differences could be found between verbal and gait performances before and after the TUGdt turning, it inspired us to further explore this as being potentially useful TUGdt outcome measures. 

The following outcome measures from the sub-sample (n = 22) were explored: time scores for TUG and TUGdt tests, TUGdt cost, i.e., relative time difference calculated as 100*(TUGdt time−TUG time)/TUG time [42], as well as number of animals recited in total as well as before and after the turning point, number of animals per ten seconds of TUGdt as well as SL before and after the turning point in both TUG and TUGdt.

To quantify SL, the video-data processing involved the digitalisation of the positions of the right and left heel and toe, respectively, derived from video data from the camera with a sagittal view. This analysis involved identifying images with heel ground contact, and in the image identify (digitise) the lower-posterior part of both heels in order to calculate the step length. The digitalisation and subsequent reconstruction of the *xy* coordinates of the heel and toe positions were performed using the software SkillSpector Version 1.3.2 (Video4Coach, Odense, Denmark). The reconstruction was based on the direct linear transformation using known positions of markings on the floor, i.e., a square of two metres (parallel to the gait direction) × 1.45 m (across the gait direction) in the middle of the three-metre walkway of the TUG set up. This method for extraction of SL from the video recordings required extensive manual work and took 40–60 min per TUGdt test trial.

#### 2.5.3. Statistical Analyses

To assess the reliability of the data processing for the digitalised SL, a testing procedure was carried out by analysing the same step (taken from the middle of the TUGdt performance) three times for each participant. This procedure was repeated three times for one chosen step in each video so that the reliability of this procedure could be evaluated. From these measurements, the intra-assay coefficient of variation (CV) was calculated.

Descriptive statistics presented as frequencies and median (minimum–maximum) values were calculated. Due to the small sample size, we used non-parametric methods for the analysis of the median with minimum and maximum values for the description. Comparisons between SL and the number of named animals before vs. after turning were performed for the whole study group using the Sign Test. Comparisons between groups were made with Willett’s residual method [43] and adjusted for age, gender, and height. Correlations were estimated as Spearman partial correlation coefficients and adjusted for age, gender, and height.

## 3. Results

### 3.1. Identification of Possible Refinements of the Study Protocol and Assessment Procedures

Feasibility of the assessment procedures was found to be good as no critical events occurred and all participants were able to complete all the tests, though some of them received complementary TUGdt instructions, e.g., if they stopped walking. On the other hand, the systematic analysis of the TUGdt video recordings revealed that deviant mobility or (more seldom) verbal performances were common in the entire group (21 out of 43), particularly among those with DD (15 out of 25). Examples of TUGdt video recordings (filtered to preserve personal integrity) includes one person with MCI (Supplementary Video S1: Woman with Mild Cognitive Impairment) and one with an established dementia diagnosis (Supplementary Video S2: Woman with Dementia Diagnosis), the latter of whom became quiet, stopped and repeatedly stood still when she conducted her TUGdt test, despite being instructed to prioritize the mobility task (see Supplementary Video S2: Woman with Dementia Diagnosis). Video analysis results (summarised in Table 2A) provided a basis for the refinement of the verification protocol for the purpose of future video analyses (Table 2B) as well as improvements of the instruction protocol for the TUG tests. The purpose of the latter was to make the instructions more precise, e.g., by including in which situations and how to use cueing, feedback, and repetition (Appendix A). It was estimated that the entire data collection procedure, in terms of tests, did not take more than 30 min/participant. Taking the participants’ status with possible fatigability into account, there was still space for completions of the UDDGait protocol with more assessments as suggested by the expert group (see interpretations and development of the UDDGait protocol below).

### 3.2. Explorations of the TUGdt Outcome Measures

The assessment of the reliability of the digitalised SL showed a mean intra-assay CV of 0.69% (range 1.4%–2.3%) and a range of SL of 0.26 to 0.73 m, which was judged as satisfying.

The statistical analyses of the sub-sample’s (n = 22) results showed that the DD group had significantly lower MMSE scores (median 20.0 vs. 27.5, p = 0.05) than NoDD. Comparisons between these groups showed no significant differences in any of the time scores of TUG, TUGdt, TUGdt cost, or named animals per ten seconds (Table 3). Significant (p < 0.005) differences were only shown for the number of animals named after the TUGdt turning point and SL difference after/before the TUGdt turning point. After the TUGdt turning point, the NoDD group was able to name significantly more animals than the participants of the DD group (Md 3.5 vs. 1.0, p < 0.05). However, no group difference was found in the number of named animals before turning or for the entire TUGdt test (Table 3).

**Figure 2:**
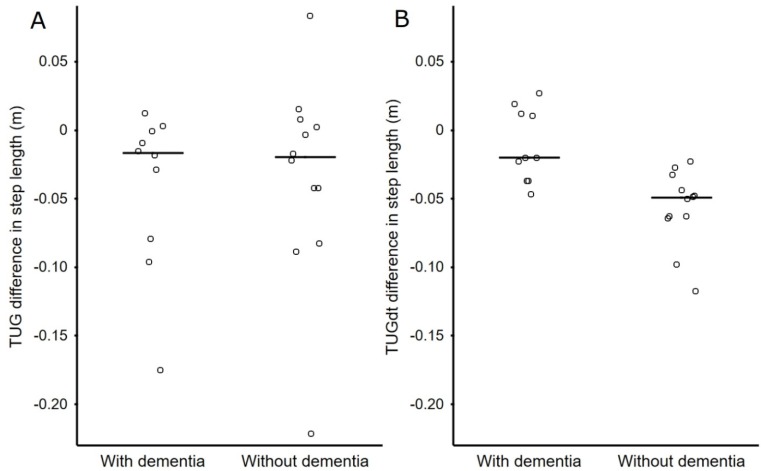
Difference in step length after vs. before turning in Timed Up-and-Go (**A**) and Timed Up-and-Go dual-task (**B**) tests among individuals with a dementia disorder diagnosis and no established dementia disorder diagnosis. Horizontal lines on the graphs show median value.

In this study sample, SL was negatively correlated with age, which was shown for both TUG and TUGdt tests (r = −0.63 to −0.68, p < 0.003). The average SL for TUG (Md = 0.402 m for the DD group and 0.555 m for the NoDD group) appeared longer than the average SL for TUGdt (Md = 0.382 m for the DD group and 0.538 m for the NoDD group) in both groups (see Figure 2). After adjustments for age, gender, and height, there was no difference shown in average SL between the groups in the total TUG or TUGdt performances. However, it was observed that the majority of participants altered their TUG test performances after turning by taking shorter steps in both TUG and TUGdt (Figure 2 and Table 3). In the whole study sample, SL was significantly shorter after turning than SL before turning in TUGdt (p = 0.006). The same tendency was also observed in the TUG test (p = 0.055). Furthermore, the number of animals named in TUGdt was significantly lower after turning (p = 0.01). Significant group differences were shown concerning SL change before vs. after turning in TUGdt (p = 0.031), but not in TUG (Figure 2). Notably, all individuals with NoDD shortened their SL after turning in TUGdt, whereas results varied in the DD group (Table 3). In TUG single-task SL, outcome variation between individuals was high in both groups (Figure 2).

### 3.3. Interpretations of Pilot Results and Development of the UDDGait Protocol

The analyses of the video-recordings, as well as recurrent consensus discussions with the interdisciplinary expert group, gave opportunities for feedback and ideas of improvements of the study protocol, its procedures, and choice of outcome variables. Based on the overall results, the following improvements were made to enable a more comprehensive description of participants the protocol was completed with three standard clinical cognitive assessment results, i.e., the Trail Making Test A and B, the Verbal Fluency Test [37] (collected from the medical records) and a depression screening using a short version of the Geriatric Depression Scale [44], as well as a balance test [45]. These were incorporated into the protocol as a complement to assessments of hand grip strength of the dominant hand using a dynamometer, the General Motor Function Assessment Scale [36], the Mini Mental State Examination (MMSE) [8], and the Clock Test [37]. Also, another TUGdt test—TUG combined with the task of reciting the months of the year in reverse order—was added, which was thought to be a more challenging task than naming animals. It places demands on declarative memory and working memory and has been shown to possess a significant diagnostic classificatory power regarding individuals with mild AD, MCI, and SCI [46]. Besides our TUGdt animals and TUGdt months, different types of cognitively challenging tasks (e.g., naming items, calculations, reciting the alphabet) have previously been paired with a mobility task to form dual-task tests. However, there is still no consensus concerning what tasks to combine to accomplish an optimal dual-task test and these tasks should not be viewed as interchangeable [24].

Additionally, refinement of the checklist for a more detailed data collection protocol was performed, including instructions of TUG and two types of TUGdt (Appendix A). A new verification protocol was developed for the examination of TUGdt-video recordings to determine which performances could be judged as normal vs. deviant (Table 2B). Establishment of blinded (in relation to diagnosis) data collection and processing of all clinical tests will be applied as a rule in the UDDGait protocol.

In an effort to catch most of the potential of the TUGdt outcome, we explored some novel and rarely used measures including verbal performance (number of animals recited) and a gait parameter (SL) before and after the TUGdt turning point, as well as number of animals per ten seconds, which were calculated alongside the more established time scores. It is to be noted that although dual-task testing consists of a combination of two tasks—most commonly a verbal and a mobility task—the verbal performance has rarely been reported, though words per ten seconds during usual pace gait have been recorded in one previous study [22]. Thus, the full potential of the test results may not have been completely considered in research. Given the small sample size, which was consistent with our aim directed towards protocol development, all the explored TUGdt outcome measures should be of interest for further examination in more extensive UDDGait investigations.

#### 3.3.1. The Refined Protocol for the Large-Scale UDDGait Study

**Figure 3:**
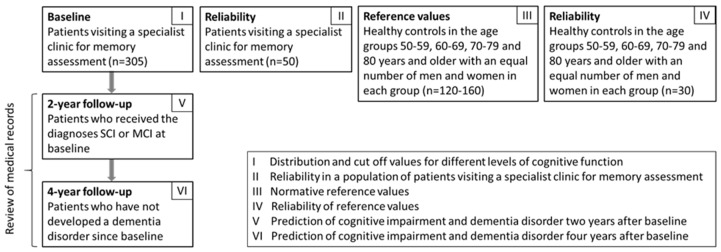
Overview of planned and initiated UDDGait-studies I–VI.

The UDDGait is designed as a prospective cohort study with experimental elements, focusing on the analysis of if and how TUGdt outcomes can predict cognitive decline and dementia disorders. It includes investigations of reliability and normative reference values of TUGdt outcomes (Figure 3).

Patients attending a specialist clinic for memory assessments at the Uppsala University Hospital or the Falun Regional Hospital were recruited before they are diagnosed and then followed over a four-year period. Inclusion criteria for this population: Patients who undergo memory testing at specialist clinics. Exclusion criteria: difficulties in carrying out verbal instructions given in Swedish (due to, e.g., restrictions for vision/hearing or needing an interpreter), health problems that significantly limit the ability to walk, indoor use of a walker, and the inability to get up from a sitting position. For the identification of cut-off values of TUGdt results for individuals with different levels of cognitive functioning (SCI, MCI, and dementia diagnosis) and for the longitudinal prospective sub-studies (Studies I, V and VI; Figure 3), 305 patients were recruited in accordance with the statistical power analysis presented below. Follow up assessments were performed two and four years after the baseline with patients who had not developed a dementia disorder until this time and the medical records from all these individuals will be reviewed mainly to identify any new cognition related diagnoses. Due to the ethical approval, we have an additional opportunity to follow these patients through their medical records for another four years. For the reliability test among patients visiting a specialist clinic for memory assessment (Study II), 50 patients will be included to enable a sub-group analysis (Figure 3).

For Study III, research participants without cognitive impairment will be recruited through advertising and community-based organisations for retirees. We are planning to include 15–20 individuals from each gender group (female/male) in the age groups 50–59, 60–69, 70–79 and 80 and older, i.e., a total of 120–160 research participants. Inclusion criteria: no experienced memory problems or self-reported severe mobility limitations. Exclusion criteria are the same as above, with the addition of MMSE <27. For the reliability test in this population (Study IV), 30 participants will be included (Figure 3).

The data collection for all participants follows the same procedure (see Section 3.3), and will be repeated for the reliability testing and longitudinal investigations. The TUGdt outcome measures described above (Section 3.2) will be further investigated.

#### 3.3.2. Statistical Power Calculation for the UDDGait-Protocol

Our starting point was that TUGdt cost (the most established dual-task outcome) can demonstrate significant differences between those who will develop and those who will not develop a dementia disorder in the included population that undergoes memory assessments at specialist clinics. Our hypothesis is that TUGdt cost has a predictive capacity with a c-statistic of ≥0.8. As such, 198 patients are required in order to attain a 90% probability (power) that the lower confidence limit is >0.7, presuming that 1/3 of the patients will develop dementia within three years (based on data from previous studies). Our presumption that c >0.7 is clinically relevant: i.e., that approximately 70% of the predictions will prove to be correct. So as to compensate for an expected proportion of 35% who will demonstrate a dementia diagnosis at the baseline measurement stage, 305 patients are required for inclusion. For each of the reliability studies, we aim to evaluate 30 and 50 participants/patients, respectively. The larger population has the purpose of enabling a subgroup analysis for patients that receive the MCI diagnosis. We assume that ICC for TUGdt cost is >0.85, and this implies that the length of a 95% confidence interval for ICC is <0.10.

## 4. Discussion

To the best of our knowledge, this study is the first to examine the feasibility of dual-task testing in a non-laboratory clinical setting among individuals undergoing memory assessments, using systematic analyses of video-recorded tests. This implies that we report on the preparatory processes, including piloting and feasibility evaluation, for the development of a large-scale study, which corresponds to inquiries for this kind of publication. It has been claimed that it is important to meet the need for more such publication [47], also concerning non-randomised studies since reporting can be beneficial for researchers in learning across disciplines, reusing techniques and avoiding repetition of similar pitfalls. [34] However, uncertainty still remains in how to report such work [34]. The only previous dual-task feasibility study found was laboratory-based and included participants with a dementia diagnosis [48]. It included tests conducted on an instrumented walkway with usual walking combined with various verbal tasks (including naming animals). Both walking and talking were, in contrast to our TUGdt, supposed to be performed as fast as possible without prioritizing one of them [48]. Such instructions may be experienced as stressful for a population undergoing memory assessment and the response may not be indicative of current functional status [49]. Nevertheless, it was indicated that such dual-task tests could be suitable as a diagnostic or descriptive tool for cognitive decline [48].

The current feasibility analysis with a video-based methodology enabled systematic and repeated observations of both mobility and verbal performances for the discovery of uncertainties concerning the interplay between test administration and participants’ responses to the assessment instructions and validation of the data collection. This kind of information is seldom presented in research [24]. The only dual-task study found in which the quality of participants’ performances was analysed; performances analyses categorised performances as normal, moderate deviation, or severe deviation, which was apparently based on observations at the same time as the testing occurred [50]. In that study, it was found that 98% of healthy controls, 62% of those with MCI, and 35% of those with Alzheimer’s disease were categorised as having normal performance, which emphasizes the importance of paying attention to and managing these phenomena to improve the test validity. Moreover, categorisation based on cautious analyses of TUGdt performances might be used as an outcome measure in its own right, since deviant performance can be associated with the progression from MCI to dementia [29]. It has been suggested that the qualitative evaluation of dual-tasking may outperform quantitative measures when it comes to differentiating between progressive and stable MCI [50], but this hypothesis still needs further investigation. It is preferable for such analyses to be based on video-recorded tests to ensure a high degree of reliability.

As shown by our video analyses, deviant performances occurred in all the TUG sub-sequences: initiation and rising up, walking forward, crossing the line and turning back, and walking back and sitting down (Table 2A). One participant with DD recurrently stopped both talking and walking during the TUGdt test (see Supplementary Video S2: Woman with Dementia Diagnosis), which might be a sign of the test not being the best suited for individuals with moderate to severe dementia disorders. However, it appears to be executable for individuals with less severe cognitive impairments and hence suitable to work as a screening tool for the prediction of dementia incidences. The identification of deviant performances provided a basis for the development of the UDDGait data collection protocol concerning the improved specification of test instructions, including the standardisation of reasons for and how to use cueing, as well as when feedback and test repetition are to be used. These specifications should decrease the risk for both longer durations of stop-walking-while-talking [51] occurrences and unstructured encouragement, and thereby improve the assessment validity [52]. This is also in line with previous research arguing that it is critically important to advance the use of assessments of the cognitive motor interference by dual-tasking and that the use of a more standardised approach that allows comparisons is urgent in both research and clinical settings [41]. Such standardisation with the presentation of specified instructions may be of particular importance when TUG is used for dual-task testing, as it is a more complex mobility task than using ordinary gait alone.

In line with guidelines concerning pilot and feasibility study aims [47], our exploration of TUGdt outcome measures was not designed or powered to address hypothesis testing. This is an objective for a large-scale UDDGait study. In contrast, the current exploration was aimed at evaluating the procedures for data collection, preparation and analyses and at indicating selection of the most appropriate outcome measure (s). It was therefore not surprising that we did not find any differences between the DD group and the NoDD group in any of the temporal outcomes, including dual-task cost or in the number of animals per ten seconds. Despite this, thought-provoking results from the current study revealed that the NoDD group named significantly more animals than participants in the DD group after turning: 3.5 vs. 1.0. Additionally, a significant difference was found concerning a change in SL after the turning point in TUGdt (but not in TUG), explained by the fact that all individuals in the NoDD group shortened their SL after turning, whereas these results varied in the DD group. These results correspond to some of the results from a recent study, in which an instrumented walkway was used for an extended (ten meters) version of the TUG test [53]. In that study, four gait parameters other than SL, namely, velocity, stride velocity, and the proportion of the double support phase with respect to gait cycle duration, showed a statistically significant difference between gait for walking away and walking back in persons with Parkinson’s disease, but not in healthy controls. Hence, it appears that our comparisons of SL generated some interesting results. Data extraction of SL from video-data was, however, time-consuming.

All of the above outcome measures are considered to be feasible and potentially appropriate for future investigations in a larger sample in the large-scale study. The time-related outcomes are interesting since they are the most established outcomes from dual-task testing and may, hence, provide opportunities for comparisons with other research results. Additionally, an UDDGait sub-study of 90 participants (age range 49–84 years) undergoing memory assessment showed that the number of correct animals named, as well as the number of correct animals named per ten seconds during TUGdt, correlated with biomarkers for AD (t-tau and p-tau) [54] and that preliminary UDDGait baseline results [55] have also indicated that TUGdt outcomes “words per time unit”, i.e., “animals per 10 s” and “months per 10 s” can discriminate between groups of individuals with early-stage dementia diagnoses, MCI, SCI, and healthy controls.

The SL and other step parameters are interesting but a large sample would presumably require a more semi-automated system with less manual elements, which would open the way for the examination of more spatiotemporal gait parameters. Such a development has the potential of being fruitful, as research results have indicated that specific gait characteristics derived from dual-task tests (and usual pace walking) in a preclinical stage and in MCI, are associated with the risk of developing specific dementia types [27]. We therefore aim for the development of a system that can extract gait parameters from video recordings, based on deep-learning methods [56] as a UDDGait side-project.

## 5. Study Limitations and Strengths

This study has a number of limitations that should be taken into consideration when interpreting the results. The pilot sample size was quite small, which implies a risk of missing possibilities of discovering uncertainties concerning the interplay between TUGdt administration and participants’ performances as well as true group differences, which in turn limits the possibility for generalizing the results. Moreover, the grouping of participants can be questioned since three individuals of the NoDD group, at the time of the data collection, were involved in an ongoing process of memory assessment with unknown final results. On the other hand, the group differences of MMSE scores (Table 1) indicate that the NoDD and DD groups differ in degree of cognitive impairment. Strengths of the methodology used include the use of video-recordings that enable cautious and repeated observations and analyses of the TUG and TUGdt performances and the reliability test of the processing for achieving SL data from the video recording, which showed satisfying results.

There are several advantages to using video recordings: the equipment is inexpensive, assessable and does not require a large amount of space, which makes it useful in a clinical environment. Moreover, these recordings allow for the simultaneous data collection of mobility and verbal performances and, if desired, it can also be used for collecting data on timing. However, the extraction of gait parameters in an effective way suitable for clinical practices will require the development of a system that provides this in a reliable, fast and semi-automatic manner. Since the long term goal of this pilot study is to provide results that can guide the design of more extensive studies including later clinical implementation studies, the clinically-based approach with the use of transportable and inexpensive equipment and with a combination of well-established and easily performed test procedures may also be viewed as a strength. 

## 6. Conclusions

To the best of our knowledge, this is the first study that examines the feasibility of TUGdt testing through the use of systematic analyses of video-recorded tests in a clinical setting. Additionally, our exploration of TUGdt outcomes involved some novel measures, such as the number of words (animals) recited per ten seconds and numbers of words and SL before and after the turning point in TUGdt. Despite some deviant TUGdt test performances identified through the videos, the test procedures are judged to possess the potential for good feasibility after protocol refinements were made. The TUGdt outcomes showed some promising results by indicating that changes in SL and possibly also the number of words correctly recited during TUGdt testing could be relevant parameters to investigate as a marker for dementia. Follow-up studies with larger samples focused on the investigation of these parameters in populations with sub-groups of individuals with different degrees of cognitive functioning, including healthy controls, are being carried out to provide a basis for possible clinical implementation of the TUGdt test.

## Figures and Tables

**Table 1:** Overview of the total study sample (n = 43) and the sub-sample (n = 22).

	Dementia Diagnosis	No Dementia Diagnosis	All
**Total sample (n)**	25	18	43
Age (years), Md (range)	77 (59–87)	72 (58–83)	74 (58–87)
Female n, (%)	10 (40)	7(38)	17 (37)
MMSE (scores), Md (range)	22 (20–24)	26 (21–29)	22 (20–27)
**Sub-sample (n)**	10	12	22
Age (years), Md (range)	78 (62–87)	70 (58–76)	71 (58–87)
Female n, (%)	4 (40)	6 (50)	10 (45.5)
MMSE (scores), Md (range)	20.0 (15–27)	27.5 (19–30)	26.0 (15–30)

MMSE = Mini Mental State Examination, Md = Median.

**Table 2:** Overview of results from qualitative analysis of video recorded Timed Up-and-Go dual task test performances and the verification protocol developed through interpretation of these results.

Mobility-	A) Observations of Deviant Performance		B) Verification Protocol (Based on A)	
Sequence	Mobility	Verbal	Normal Performance	Deviant Performance
**Initiation and rising up from the chair**	Starts during the instructions Rises and stands still/ and asks: Shall I start at once?	Asks: Shall I start with the animals?/Shall I say it out loud?	Starts after finished instructions Rises and starts walking	Asks about the verbal and/or mobility performance at the start
**Walking 3 m to the line**	Stops walking Stops walking before the line Stops after the turn Asks: Shall I turn?	Tells a story about animals Asks: Shall I talk about the animals now?	Walks without any stops	Stands still >1 s.
**Line crossing and turning**	Turns before the line/with one foot on/two feet on the line Wide turn Walks (2 m) up to the camera before turning Asks: Shall I turn here?		Passes the line. Turns with at least one foot over the line	No foot over the line More than two stances (i.e., more than one step) before the turn is initiated Stands still >1 s.
**Walking back 3 m to chair**	Stops walking	Asks: Shall I say the same thing now?	Walks without any stops to the chair and turns (to sit down)	Stands still >1 s.
**Sitting down on chair**	Walks to/past the chair and stands still Asks: Shall I sit down? / Is this enough?		Sits down spontaneously	Does not spontaneously sit down Walks past the chair
**General**	Discontinues the test	Says nothing, i.e., no animals Small talk	Names animals Verbal hesitation or correction of words Small talk combined with naming animals	Says nouns that are not animals Says nothing Discontinues the test Asks questions about the mobility or verbal tasks during the test

**Table 3:** Results of Timed Up-and-GO (TUG) and TUG dual-task (TUGdt) test measurements in patients with dementia disorder diagnosis and no established dementia diagnosis. Significant *p*-values in bold font.

Variable	With Dementia (N = 10)			No Dementia (N = 12)			Adjusted *p*-Value *
	Min	Md	Max	Min	Md	Max	
TUG time (s)	10.4	18.1	30.2	7.5	14.4	19.4	0.342
TUGdt time (s)	13.9	28.7	55.0	10.2	17.6	41.9	0.149
TUGdt cost (%)	15.3	37.8	217.6	1.0	27.3	115.9	0.972
TUGdt animals before turning (n)	1	3	7	0	4	6	0.418
TUGdt animals after turning (n)	0	1	3	0	3.5	5	0.045
TUGdt animals difference after/before turning (n)	−5	−2	0	−4	−1.5	5	0.418
TUGdt animals per 10 (s)	0.76	1.8	4.3	1.3	4.9	8.2	0.245
TUG SL before turning (m)	0.251	0.415	0.625	0.431	0.588	0.746	0.149
TUG SL after turning (m)	0.254	0.386	0.637	0.351	0.534	0.704	0.342
TUG SL difference after/before turning (m)	−0.175	−0.017	0.012	−0.222	−0.020	0.084	0.597
TUGdt SL before turning (m)	0.215	0.385	0.593	0.353	0.568	0.718	0.062
TUGdt SL after turning (m)	0.236	0.373	0.605	0.320	0.502	0.671	0.379
TUGdt SL difference after/before turning (m)	−0.047	−0.020	0.027	−0.118	−0.049	−0.023	0.032

* Comparison between groups using Wilcoxon’s two-sample test and adjusted for age, gender and height with Willett’s residual method; Min = minimum, Md = Median, Max = maximum; TUG = Timed Up-and-Go, TUGdt = TUG dual-task, SL = step length.

## Supplementary Materials

The following are available online at https://www.mdpi.com/1660-4601/17/5/1715/s1, Video S1: Milde Cognitive Impairment, Video S2: Dementia Diagnosis.

## Appendix A

### Appendix A.1. Data Collection of Timed Up-and-Go (TUG) and Two Types of TUG Dual Task (TUGdt): TUGdt Animals and TUGdt Months

#### Appendix A.1.1. TUG and TUGdt Tests

TUG and TUGdt tests are performed according to a standardized set up and procedures including video recordings with moving image and sound recordings. The video cameras are mounted on tripods, one with a frontal view and one with a lateral view, and synchronised for data collection.

#### Appendix A.1.2. Two TUGdt Tests

TUG combined with “name different animals” and “recite the months of the year in reverse order”, respectively. Both tasks are to be completed at the participant’s own speed and with the instruction to prioritise the walking part—i.e., if the participant is unable to say words, he/she should continue walking and continue to complete the movement sequence.

#### Appendix A.1.3. TUG Test

Preparation: Place a chair (46 cm in height) with armrests three metres from a line made by a clearly visible strip of tape on the floor, parallel to the front legs. The distance is measured from the front legs of the chair.

Starting position: The participant sits on the chair with his/her arms on the armrests and his/her back against the backrest.

Execution: The test begins with the assessor describing and showing how the test is performed (see point 2 of instructions below).

The participant should perform the following sequence of movements at his/her own comfortable pace: From sitting in a chair with armrests, get up, walk three metres, pass over the floor marking (a clearly visible strip of tape on the floor), turn around on the other side of the line, walk back and sit down in the chair.

1. Pre-trial: The participant performs the TUG test once without timing: **“You can try now”**. If necessary, supplementary instructions are given while the participant is performing the trial test.
2. TUG single-task (timed): The following instructions are given to the participant immediately before the test is performed: “When I say start, you should rise up, walk towards the line, cross the line, turn back, walk back and sit down in the chair. You should walk normally, as you usually do, at your own pace. Are you ready?” After an affirmative answer, the assessor says: “You can start now”. Timing starts when the participant’s back leaves the chair back and ends when the participant’s posterior touches the seat. Oral clues are provided if necessary and noted in the minutes (see below: IMPORTANT).
3. TUG dual-task (Animals): The following instructions are given to the participant “You should do what you just did, but at the same time as you walk, please name different animals aloud. You can choose any animals you want. If you get stuck and cannot think of any animals, just keep walking like you just did now”.
  Answer any questions.
  Ask the question: **“Are you ready?”**. After an affirmative answer, the assessor says: **“You can start now”**. The assessor times the performance. (The number of different animals is extracted from the video recordings).
4. TUG dual-task (Months): The following instructions are given to the participant “You should do what you just did, but at the same time as you walk, please recite the months of the year in reverse order. So you should start with the last month. If you get stuck and do not know what month to say, just keep walking like you just did now. You don’t have to recite all of the months”.
  Answer any questions.
  Ask the question: “Are you ready?”. After an affirmative answer, the assessor says: “You can start now”. The assessor times the performance. (The number of months in correct order is extracted from the video recordings).

### Appendix A.2. Important Considerations

The starting point is when the participant knows how the TUG-single task test is to be performed.

Repeat the instructions if needed and clarify them if necessary. Supplement with a new demonstration if needed. Note extra instructions in the protocol.

If the participant asks what to do immediately after rising up from the chair when the test is initiated, cancel. Provide renewed instructions and a demonstration and restart. Restart is allowed only once.

The following oral clues can be given to participants during the ongoing test and should be noted in the protocol. Gestures, e.g. to invite the participant to sit on the chair, are noted as “clue”.

1. Briefly answer questions about the execution.
2. Give the instruction “turn”, if the person does not turn when passing the floor marking but walks straight ahead towards the camera in front.
3. Give the instruction “sit down” if the person on the way back stops next to the chair and remains standing.
4. If the participant slows down or makes short stops (≤10 s) at other times during the test, no encouraging instructions are given.
5. In cases where the person has obviously forgotten what to do, or expresses that s/he cannot perform the test, encouragement to continue walking is given.
